# The Paternal Transition Entails Neuroanatomic Adaptations that are Associated with the Father’s Brain Response to his Infant Cues

**DOI:** 10.1093/texcom/tgaa082

**Published:** 2020-11-04

**Authors:** María Paternina-Die, Magdalena Martínez-García, Clara Pretus, Elseline Hoekzema, Erika Barba-Müller, Daniel Martín de Blas, Cristina Pozzobon, Agustín Ballesteros, Óscar Vilarroya, Manuel Desco, Susanna Carmona

**Affiliations:** 1 Instituto de Investigación Sanitaria Gregorio Marañón, 28007 Madrid, Spain; 2 Centro de Investigación Biomédica en Red de Salud Mental (CIBERSAM), 28029 Madrid, Spain; 3 Institut Hospital del Mar d’Investigacions Mèdiques, 08003 Barcelona, Spain; 4 Departament de Psiquiatria i Medicina Legal, Universitat Autònoma de Barcelona, Cerdanyola del Vallés, 08193 Barcelona, Spain; 5 Brain and Development Research Center, Leiden University, 2333 Leiden, the Netherlands; 6 Leiden Institute for Brain and Cognition, 2300 Leiden, the Netherlands; 7 Institute of Mental Health Vidal i Barraquer, Ramon Llull University, 08022 Barcelona, Spain; 8 Instituto Valenciano de Infertilidad, 20145 Milan, Italy; 9 Instituto Valenciano de Infertilidad, 08017 Barcelona, Spain; 10 Departamento de Bioingeniería e Ingeniería Aeroespacial, Universidad Carlos III de Madrid, 28903 Madrid, Spain; 11 Centro Nacional de Investigaciones Cardiovasculares Carlos III, 28029 Madrid, Spain

**Keywords:** fatherhood, MRI, neuroimaging, parental brain

## Abstract

The transition into fatherhood is a life-changing event that requires substantial psychological adaptations. In families that include a father figure, sensitive paternal behavior has been shown to positively impact the infant’s development. Yet, studies exploring the neuroanatomic adaptations of men in their transition into fatherhood are scarce. The present study used surface-based methods to reanalyze a previously published prospective magnetic resonance imaging dataset comprised of 20 first-time fathers (preconception-to-postpartum) and 17 childless men. We tested if the transition into fatherhood entailed changes in cortical volume, thickness, and area and whether these changes were related to 2 indicators of paternal experience. Specifically, we tested if such changes were associated with (1) the baby’s age and/or (2) the fathers’ brain activity in response to pictures of their babies compared with an unknown baby. Results indicated that first-time fathers exhibited a significant reduction in cortical volume and thickness of the precuneus. Moreover, higher volume reduction and cortical thinning were associated with stronger brain responses to pictures of their own baby in parental brain regions. This is the first study showing preconception-to-postpartum neuroanatomical adaptations in first-time fathers associated with the father’s brain response to cues of his infant.

## Introduction

The transition to parenthood is one of the most transformative experiences of an adult’s life, leading to adaptations at a social, psychological, and physiological level (Saxbe et al. 2018). Western societies have witnessed a recent increase in fathers’ investment in childcare (Cabrera et al. 2000; Gauthier et al. 2004; O’Brien et al. 2007). This socio-cultural shift has led to an increasing interest in unveiling the neural underpinnings of paternal behavior and their unique contributions to the child’s well-being (Sarkadi et al. 2008; Abraham et al. 2014, 2016; Malmberg et al. 2016). These research questions may be relevant for family policies such as parental leave provisions for fathers (Feldman et al. 2019).

As compared with maternal care, paternal care appears less frequently across species and is more variable within individuals of the same species (Geary 2015). Paternal behavior is observed in only 5% of mammalian species (Woodroffe and Vincent 1994; Dulac et al. 2014), and males’ behaviors range from avoidance or even aggression towards the infants to direct parental engagement (Dulac et al. 2014). In humans, paternal investment is affected by socio-cultural (Doherty et al. 1998; Ozgun et al. 2011) and historical differences (Rohner 1998; Sayer et al. 2004), as well as individual differences, producing behaviors that range from a father being fully absent to being the primary caregiver.

Behavioral variability in the expression of paternal care is reflected in men’s hormonal and neural systems. For instance, fathers who spend more daily time with their children show lower levels of testosterone (Gettler et al. 2011), whereas fathers with more exploratory and affectional parenting behaviors exhibit higher levels of prolactin and oxytocin, respectively (Gordon et al. 2010a, 2010b). Moreover, functional magnetic resonance imaging (MRI) studies have shown that fathers, similarly to mothers, activate regions of the default mode network in response to their infant cues (Leibenluft et al. 2004; Atzil et al. 2012; Abraham et al. 2014). These regions have been extensively involved in empathy and prosocial behaviors not only in parents but also in the general population (Li et al. 2014; Feldman et al. 2019). Notably, this pattern of neural activation has been associated with the time that fathers reported spending with the child. Specifically, the more weekly hours a father spent alone with his infant, the higher the functional connectivity between default mode and reward system regions (Abraham et al. 2014).

To date, it is still unclear if fatherhood also modifies brain anatomy. There are only 2 structural MRI studies that followed men prospectively during their transition into fatherhood. One of them examined the postpartum period, tracking changes between 2 sessions at 2–4 and 12–16 weeks postpartum (Kim et al. 2014); the other study, which was published by our group, included the pregnancy period by tracking changes between preconception and at approximately 10 weeks postpartum (Hoekzema et al. 2017). Whereas the first study found significant postpartum gray matter volume changes within the default mode and reward systems, the latter found no significant volume differences before and after pregnancy (Hoekzema et al. 2017). However, the latter null effects could have resulted from the high intersubject variability that characterizes paternal behavior in humans. In the present study, we reanalyzed these data using different methods to establish whether the findings of Hoekzema et al. (2017) indeed indicate (1) that the brain remains static during the transition to fatherhood or (2) that there are neuroanatomical changes related to the paternal experience.

We built on the previous voxel-based morphometric study of Hoekzema et al. (2017), reanalyzing the structural magnetic resonance (MR) brain images of first-time fathers who were scanned before and after their partners’ pregnancy. For the current study, we used a surface-based approach instead, which allowed us to separate gray matter cortical volume (CV) into cortical thickness (CT) and cortical area (CA). We used FreeSurfer registration techniques as they provide more accurate cortical estimations (Reuter et al. 2010). We also applied statistical methods that combine threshold-free cluster enhancement (TFCE) and permutation testing to improve the balance between the specificity and the sensitivity of our tests (Greve and Fischl 2019; Noble et al. 2020). Our main goal was to test whether first-time fathers’ gray matter undergoes significant changes in CV, CT, and CA. We then tested if morphometric changes in fathers were associated to 2 indicators of the paternal experience: (1) the accumulated time that fathers had spent with their infant (as estimated by the baby’s age) and (2) the fathers’ brain functional brain activation in response to visual stimuli of their baby compared with an unknown baby. The functional brain activation of a parent in response to baby stimuli has been extensively used in the parental brain literature as an indicator of parenting thoughts and behaviors (Swain 2008). This study is the first to show preconception-to-postpartum neuroanatomical adaptations in first-time fathers associated with the father’s brain response to his infant cues.

## Methods

### Participants and Longitudinal Design

The present study analyzed the prospective MR brain data of 20 first-time fathers {mean age [standard deviation (SD) = 35.60 (4.25) years]} and 17 childless men [mean age (SD) = 32.07 (6.32) years] without plans of becoming fathers in the next year. These participants were recruited and scanned as control groups for a previous longitudinal study aimed to examine the effects of pregnancy on the mother’s brain (Hoekzema et al. 2017). For every subject, anatomical MR images were acquired at 2 time points. First-time fathers were scanned before their partner’s pregnancy at the preconception level (PRE session) and again during the early postpartum [POST session; mean interscanner time (SD) = 453.75 (116.72) days; mean (SD) = 70.25 (49.21) days after delivery]. A total of 17 childless men were scanned at a time interval comparable with that of the fathers-to-be group [mean interscanner time (SD) = 419.176 (93.176) days]. In the postpartum session, fathers also participated in a functional MR task aimed to examine the neural response to visual cues of their babies as compared with the visual cues of unknown babies. Males with a self-reported history of neurological or psychiatric impairment, substance abuse disorders as well as males who were already fathers were excluded from the study. See Supplementary Figure S1 for a schematic representation of the experimental study design.

**Figure 1:**
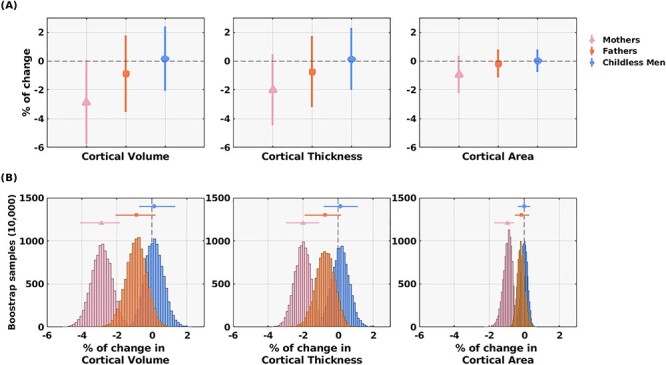
Global cortical percentages of change in first-time mothers (*N* = 25), first-time fathers (*N* = 20), and childless men (*N* = 17). (*A*) The *y*-axis represents the mean of the percentages of change. The 3 cortical measures—total cortical volume, mean cortical thickness, and total cortical area—are indicated in the *x*-axis. Vertical dispersion bars and the symbols represent the standard deviations and the mean values, respectively. (*B*) Bootstrap histograms of the mean percentage of change of each cortical measure. The *y*-axis indicates the number of bootstrap samples (10 000 random samples), and the *x*-axis indicates the mean total percentages of change of each bootstrap sample. Horizontal dispersion bars and the symbols represent the 95% confidence intervals and the mean of each bootstrap distribution, respectively. First-time mothers were included for visualization and comparative purposes.

For visualization purposes, when displaying the global percentage of change (Fig. 1), we included the data of the 25 first-time mothers [mean age (SD) = 33.85 (3.88) years] that were scanned before and after their pregnancies [mean interscanner time (SD) = 463.52 (108.33) days] in Hoekzema et al. (2017). Note that these mothers were partners of the fathers of the current study.

Table 1 provides a general description of the groups at baseline. Further details about the sample are described in Hoekzema et al. (2017).

**Table 1:** Sample description at the preconception session (baseline)

**Characteristics**	**First-time fathers**	**Childless men**	**First-time mothers**
Sample size [number of subjects]	20	17	25
Mean (SD) age [years]	35.60 (4.25)	32.065 (6.32)	33.85 (3.88)
Age range [years]	27.07–45.77	24.43–44.00	26.73–40.81
Mean (SD) interscanner time [days]	453.75 (116.72)	419.176 (93.18)	463.52 (108.33)
Mean (SD) time between parturition and postpartum session [days]	70.25 (49.21)	×	73.56 (47.83)
Education [number of subjects] School College University	2 4 14	1 3 13	2 4 19
Type of conception [number of subjects] Natural Fertility treatment	6 14	× ×	8 17
Mean (SD) total brain volume [cm^3^]	1164.60 (70.62)	1137.21 (102.69)	1008.82 (87.14)

Note: Analysis of variance *F*-tests did not show any significant difference among the 3 groups in age at preconception (*F*-value = 2.53, *P*-value = 0.088) and interscanner time (*F*-value = 0.90, *P*-value = 0.411). Age at preconception did not differ between fathers that conceived naturally or through fertility treatment (*t*-statistic = 1.53, *P*-value = 0.145). Also, first-time fathers and mothers did not differ in the time between parturition and postpartum session (*t*-statistic = 0.23, *P*-value = 0.821). There were significant group differences in the total brain volume at preconception (*F*-value = 20.70, *P*-value = 0.0001). Specifically, total brain volume differed between mothers and fathers, and between mothers and childless men [both *P*-value < 0.0001, Tukey’s Honest Significant Difference (HSD) corrected] but not between fathers and childless men (*P*-value = 0.608, Tukey HSD corrected). Pearson’s chi-squared test did not show any significant group difference neither in education (χ^2^ = 0.36, *P*-value = 0.985) nor in type of conception (χ^2^ = 0.021, *P*-value = 0.886).

Participants were recruited throughout the Instituto Valenciano de Infertilidad (Barcelona, Spain), as well as by flyers and word of mouth. The study was approved by its corresponding local ethics committee, Comitè Ètic d’Investigació Clínica de l’Institut Municipal d’Assistència Sanitària, and after a detailed explanation of the study, signed consent was obtained from all participants.

### MRI Image Acquisition

#### Structural Image Acquisition

For each subject, a high-resolution structural MRI image was acquired at preconception and postpartum sessions in a 3 Tesla Philips scanner using a *T*_1_-weighted gradient echo pulse sequence in the axial plane. The image acquisition parameters were as follows: repetition time (TR) = 8.2 ms, echo time (TE) = 3.7 ms, voxel size = 0.9375 mm × 0.9375 mm × 1 mm, field of view (FOV) = 240 mm × 240 mm × 180 mm, matrix size = 256 × 256 × 180 voxels, no gap, and a flip angle (FA) = 8°.

#### Functional Image Acquisition

First-time fathers underwent a functional MRI (fMRI) task during the postpartum session, using a *T*_2_*-weighted gradient echo-planar imaging sequence. The acquisition parameters of the 4D data were as follows: voxel size = 1.8 mm × 1.8 mm × 4 mm, acquisition matrix = 128 × 128 × 30 voxels, TR = 3000 ms, TE = 35 ms, FOV = 230.4 mm × 230.4 mm × 120 mm, gap = 0.5 mm, FA = 90°, acquisition time = 9.55 min, and the number of acquired volumes over time = 191. The fMRI paradigm examined the father’s brain’s response towards their babies compared with the brain’s response towards an unknown baby. During the experiment, first-time fathers were shown 28 pictures of their baby and 28 pictures of an unrelated, unknown baby. Pictures appeared in an event-related fashion and with a randomized order, with the following parameters: trial duration = 1500 ms, randomized intertrial interval = 750–1250 ms, and a mean number of trials (SD) = 71.3 (11.67) for their own babies and 73.40 (10.84) for unknown babies’ pictures, respectively. Pictures were sad (crying) and neutral faces expressions extracted from movies recorded at a home visit a few days before the postpartum session. All babies’ pictures had the same size, resolution, and brightness, and facial expression were cutout and placed on a black background to be presented in the fMRI task through the NeuroBehavioral Systems presentation software. Four participants were not included in the fMRI analyses: 3 of them because their head motion parameters exceeded 3 mm (for translations) or 3° (for rotations) and 1 because he fell asleep during the acquisition. This led to a final sample size of 16 fathers [mean (SD) age at preconception = 35.31 (3.82) years; mean (SD) time between parturition and postpartum session = 64.38 (37.67) days].

Due to an unexpected problem in the postpartum session, the 8-channel radiofrequency head coil had to be replaced by one of 16 channels in 9 out of 37 structural MR images (7 first-time fathers and 2 childless men, and Fisher’s exact test revealed that proportions are not significantly different; *P*-value = 0.137) and in 5 out of 16 functional MR images. To ensure that our findings did not depend on the radiofrequency head coil, we repeated the main analyses considering the possible variability induced by the radiofrequency head coil (see “Supplementary Methods and Results 1”).

### Image Processing

#### Structural Image Processing

We used surface-based methods to disentangle CV into CT and CA. Specifically, MR images were processed using the FreeSurfer longitudinal workflow (version 5.3) (Reuter et al. 2012). This workflow starts with cross-sectional processing of each preconception and postpartum image, to later create an unbiased within-subject template space using information from both MR sessions (Reuter and Fischl 2011). Then, preconception and postpartum images are remapped onto the within-subject template and longitudinally processed (Reuter et al. 2010). Several processing steps such as skull stripping, bias field correction, Talairach transforms, atlas registration, gray and white matter segmentations, as well as cortical surface parcellations are then initialized with common information from the within-subject template, thus reducing the intersubject variability and increasing the reliability and statistical power of the subsequent analyses (Reuter et al. 2012). The individual surfaces were visually inspected for major topological defects and corrected if needed. These steps resulted in 3D triangular meshes for the white and pial surfaces (inner and outer surfaces of the cortex, respectively) and vertex-wise maps of CV, CT, and CA. We restricted our analysis to the cortical mantle as the estimates for subcortical regions typically involved in maternal behavior, such as the ventral striatum, are not very reliable with automatic methods such as FreeSurfer (Biffen et al. 2020).

#### Global Cortical Structural Changes

First, we aimed to obtain estimates of the global changes in CV, CT, and CA between the preconception and the postpartum sessions. Total CV was estimated as the number of voxels belonging to the gray matter segmentation multiplied by the voxel volume. Mean CT was defined as the mean of the Euclidean distances between the white and pial surfaces of all vertices, and total CA was defined as the sum of the areas of the triangles making up the pial surface. Finally, we estimated the global percentage of change relative to the baseline level (preconception session) in CV, CT, and CA.

#### Surface-Based Morphometry

To study the morphometric changes across the brain surface, every subject’s preconception and postpartum vertex-wise maps were used to compute the within-subject maps of percentages of change relative to the baseline level (preconception session). Then, all subject maps were normalized into the fsaverage to achieve a vertex correspondence among subjects (Reuter et al. 2010, 2012; Reuter and Fischl 2011). Finally, the normalized maps were smoothed with 10 iterations nearest neighbor averaging kernel [equivalent to a Gaussian kernel of 10 mm full-width half-maximum (FWHM)] to alleviate possible outliers and asymmetries and to ensure a correspondence among brain regions. We performed these steps for the hemispheric CV, CT, and CA measures separately.

#### Functional Image Processing

The fMRI images were processed in Statistical Parametric Mapping, implemented in Matlab 2017b (MathWorks, Inc.). First, images were corrected for differences in slice timing acquisition and realigned into the mean functional image to control for motion-related artifacts. Then, functional and structural images were coregistered and normalized into the Montreal Neurological Institute space. Finally, a Gaussian kernel of 12 mm FWHM was applied to the images.

### Statistical Analyses

#### Global Cortical Structural Changes

Statistical analyses of the total percentages of change were performed using Matlab R2017b. First, we fitted a general linear model (GLM) per cortical measure—CV, CT, and CA—including group membership and the age *z*-scores as covariates. Then, two-sided parametric analyses tested whether the percentage of change differed from 0 and among the groups. Results were considered statistically significant if they survived the threshold of *P*-value < 0.05 after adjusting for false discovery rate (FDR) with a *Q*-value of 0.05 (Benjamini and Hochberg 1995). Effect sizes were measured as Cohen’s *d*. As complementary analyses and to obtain more accurate estimations of the population morphometric changes, we re-estimated the basic descriptive statistics (mean and SD) and 95% confidence intervals (CIs) using bootstrap analyses. Specifically, we employed the Matlab’s “bootstrp” and “bootci” functions to draw 10 000 random samples with replacement of the total morphometric percentage of change.

#### Surface-Based Morphometry

We applied a vertex-wise GLM analysis for each cortical measure (CT, CV, and CA) and hemisphere using Permutation Analysis of Linear Models (PALM) (version alpha-115), implemented in FMRIB Software Library (version 6.0.2). We used PALM instead of the FreeSurfer cluster-wise correction method—mri_glmfit-sim function—as it reduces the rate of false positives (Greve and Fischl 2019). We examined in first-time fathers and childless men whether the morphometric percentage of change significantly differed from 0 or between the groups (i.e., greater reductions in fathers compared with the childless men and the reverse contrast). The adjusted models contained group membership and age *z*-scores as covariates. The resulting *t*-statistic maps of the different contrasts were transformed into TFCE scores using the default surface parameters *E* = 1 and *H* = 2 (Smith and Nichols 2009), and statistical significance was obtained using permutations methods (10 000 permutations) (Winkler et al. 2014). To account for the multiple testing problem, we applied family-wise error (FWE) correction using the distribution of the maximum statistic across both hemispheres (Winkler et al. 2014). We thresholded the maps of the effect sizes (Cohen’s *d*) with those results surviving the threshold of TFCE, FWE-corrected *P*-value < 0.05.

#### Functional Brain Activations

At the first-level analysis, we applied a mass univariate analysis to every subject to model the voxel-wise responses in blood oxygen level-dependent signal for the conditions of (1) “own baby> unknown baby’s pictures” and (2) “unknown baby> own baby’s pictures.” We controlled for head motion parameters extracted from the realignment preprocessing stage. Also, the temporal autocorrelation was removed through an autoregressive model of order one. First-level estimate maps were entered into a second-level analysis using the FSL’s tool Randomise (Winkler et al. 2014). We chose Randomise as it uses TFCE and permutations, thus making it a suitable tool to compare our structural and functional findings. Specifically, we examined if first-time fathers showed differences in neural activity when responding to pictures of their baby compared with pictures of an unknown baby. Neural activity differences were examined within a gray matter cortical mask based on Yeo’s functional parcellation (Yeo et al. 2011), to facilitate the interpretation of the activations in terms of large-scale functional networks. The specified model included age *z*-scores as a covariate. The resulting *t*-statistic maps were transformed into TFCE scores with parameter values of *E* = 0.5 and *H* = 2 (Smith and Nichols 2009). Statistical significance was obtained using permutations methods (10 000 sign flipping transformations), and results were FWE-corrected using the distribution of the maximum statistic (Winkler et al. 2014). Finally, we computed the effect sizes (Cohen’s *d*) of the functional brain activations.

#### Parcellation in Yeo Networks

Structural surface-based changes and functional brain activations were classified into their corresponding networks according to the functional parcellation of Yeo et al. (2011). Specifically, we computed the percentage of vertices and voxels—structural and functional information, respectively—belonging to each of the 7 cortical networks in the parcellation atlas obtained from FreeSurfer (https://surfer.nmr.mgh.harvard.edu/fswiki/CorticalParcellation_Yeo2011).

#### Correlations between Cortical Changes and Indicators of Paternal Experience

We studied if the morphometric changes in first-time fathers were related to indicators of paternal experience: (1) the accumulated time that fathers had spent with their babies (as estimated by baby’s age) and (2) the degree of brain activations upon their babies’ pictures compared with unknown baby pictures (as estimated by the mean of the significant functional brain activations). To obtain the morphological brain changes, we used the results of the surface-based morphometric analysis. Specifically, we computed the mean percentage of change within those vertices with significant decreases in fathers (TFCE, FWE-corrected *P*-value < 0.05). Then, we computed two-tailed Pearson’s correlations between the mean morphometric percentages of change and both indicators of paternal experience. Correlations were considered significant if they survived the threshold of *P*-value < 0.05 after adjusting for FDR with a *Q*-value of 0.05 (Benjamini and Hochberg 1995).

## Results

### Global Cortical Structural Changes

Figure 1 displays the percentages of change in total CV, mean CT, and total CA of first-time fathers and childless men. For all the groups, Figure 1*A* shows the mean and SDs of the percentages of change, and Figure 1*B* shows the bootstrap distributions of the mean percentage of change as estimated with 10 000 random samples with replacement. For visualization purposes, when displaying the total percentage of change (Fig. 1), we included the data of the 25 first-time mothers that were scanned before and after their pregnancies in our previous study (Hoekzema et al. 2017).

Mean (SD) percentages of change in first-time fathers were as follows: CV = −0.90 (2.66), CT = −0.75 (2.45), and CA = −0.18 (0.96). Within-group comparisons indicate that neither first-time fathers (all false FDR-adjusted *P*-value > 0.337) nor childless men (all FDR-adjusted *P*-value > 0.903) exhibited changes significantly different from 0 in cortical measures. That was not the case for first-time mothers, who showed significant reductions in all cortical measures (all FDR-adjusted *P*-value < 0.004). However, as observed in Figure 1, fathers’ mean percentages of change and bootstrap distributions of CV and CT lay between those of mothers and childless men. In fact, fathers’ and mothers’ bootstrap CIs overlapped in CV and CT, but not in CA (Fig. 1*B*). Group comparisons indicated that fathers did not differ either from mothers (FDR-adjusted *P*-values: CV = 0.053, CT = 0.145, and CA = 0.057) or from childless males (all FDR-adjusted *P*-value > 0.272) in any cortical measure, although the comparison between fathers and mothers was close to the statistical significance.

Further descriptive and inferential statistics of within-group comparisons, between-group comparisons, and bootstrap distributions are displayed in Supplementary Table S1.

### Surface-Based Morphometry

**Figure 2:**
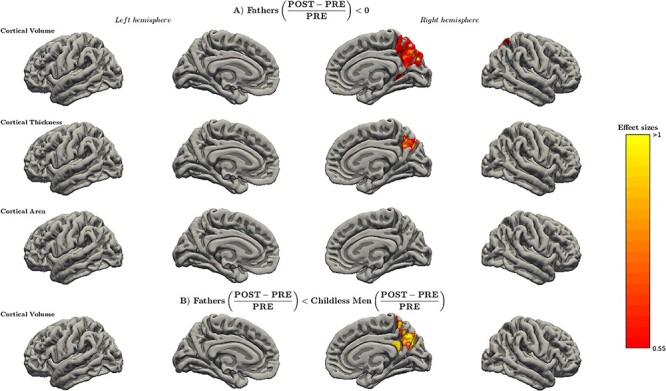
Surface-based cortical changes in first-time fathers (*N* = 20) and between fathers and childless men (*N* = 17) groups. (*A*) Reductions in first-time fathers relative to baseline (preconception session). Cohen’s *d* effect sizes range between 0.55 and 1.12 for cortical volume and between 0.71 and 1.12 for cortical thickness. (*B*) Greater volumetric reductions in first-time fathers compared with childless men. Effect sizes range from 0.80 to 1.48. The represented effect sizes correspond to the results surviving the restrictive threshold of TFCE, *P*-value < 0.05 FWE corrected. Brain surfaces correspond to the pial fsaverage template of FreeSurfer. PRE, preconception; POST, postpartum.

Figure 2 shows the surface-based within-group changes in first-time fathers and the group differences between first-time fathers and childless men in CV, CT, and CA.

First-time fathers exhibited volumetric reductions in the right precuneus in the within-group analysis (Fig. 2*A*). They also displayed decreases in CT in the same region, whereas CA did not display any significant change. Conversely, no significant increases were found in any of the cortical measures. The group of childless men did not render significant within-group changes. Between-group comparisons revealed significant CV differences between fathers and childless men (Fig. 2*B*). Specifically, fathers had more volumetric reductions in the right precuneus extending into the posterior cingulate cortex compared with childless men, whereas CT and CA did not significantly change. No significant increases were found in fathers compared with childless men. Finally, to identify the affected large-scale networks, we quantified the percentage of overlap of the surface-based results with each of the 7 functional networks described by Yeo et al. (2011). Results indicated that within-group and between-group findings mainly overlapped with the default mode network. The exact percentages of overlap are represented in Supplementary Figure S2.

### Correlations between Cortical Changes and Indicators of Paternal Experience

**Figure 3:**
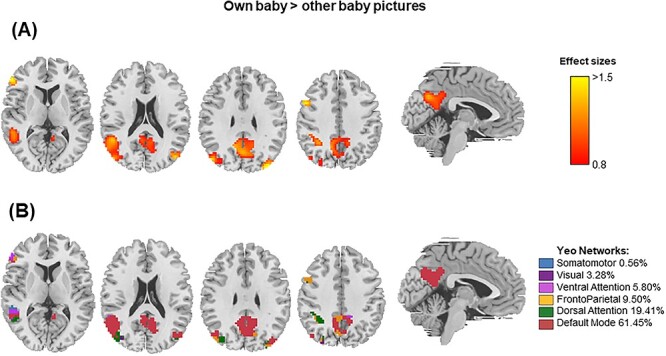
Functional brain activations in first-time fathers (*N* = 16) for the contrast “own baby> unknown baby.” (*A*) The represented effect sizes (Cohen’s *d*) range from 0.84 to 1.82 and correspond to results surviving the restrictive threshold of TFCE, *P-*value < 0.05 FWE-corrected. (*B*) Percentage of overlap between the functional brain activations depicted in *A* and each of the 7 functional networks of Yeo et al. (2011). Each network is depicted in a different color. Slices correspond to the axial views (MNI coordinates: 9, 21, 30, and 39) and the sagittal view (MNI coordinate: −3). MNI, Montreal Neurological Institute; L, left hemisphere; R, right hemisphere.

We tested if the surface-based brain changes found in fathers (Fig. 2*A*) were related to markers of paternal experience by looking into (1) the accumulated time fathers had spent with their infants (estimated by the baby’s age at the postpartum session) and (2) the fathers’ degree of brain activation in response to pictures of their own baby compared with an unknown baby (Fig. 3).

#### Baby’s Age

No significant correlations were found between the morphological brain changes and the baby’s age (*r*_CV_ = 0.07 and *r*_CT_ = 0.02, uncorrected *P*-value > 0.777).

#### Degree of Brain Activation to Own Baby Versus Unknown Baby

We analyzed the neural response of first-time fathers when presented with the face of their baby versus faces of an unknown baby. Figure 3 shows the effect sizes of the contrast “own baby> unknown baby” (TFCE, FWE-corrected *P*-value < 0.05), which rendered bilateral activations in the precuneus, middle temporal gyrus, posterior cingulate cortex, and middle occipital lobes, as well as right-sided activations in the inferior frontal/parietal gyrus, angular/precentral gyrus, inferior/posterior temporal gyrus, and superior occipital lobe. Most of the functional brain activations overlapped with the default mode and dorsal attention networks (61.45% and 19.41% of the total activations, respectively; Fig. 3*B*). The reverse contrast (“unknown baby> own baby”) did not produce any significant results.

**Figure 4:**
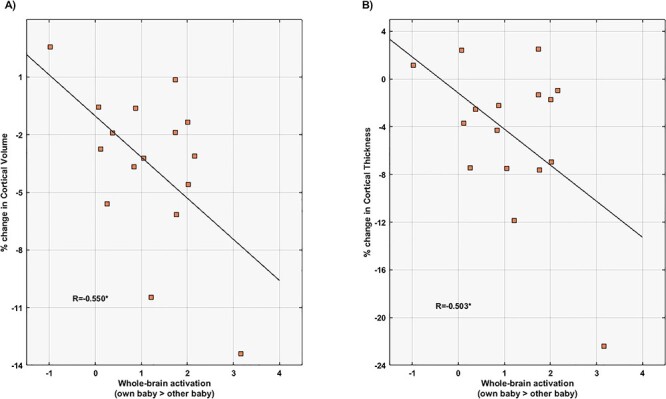
Correlations between cortical changes and functional brain activations in first-time fathers (*N* = 16). The *x*-axis represents the mean of the significant functional brain activations towards pictures of their own baby compared with an unknown baby. The *y*-axis represents the mean percentages of change in cortical volume (*A*) and cortical thickness (*B*) extracted from the surface-based morphometric analysis. Asterisks indicate the Pearson’s correlations coefficients (*r*) that survived the false discovery rate-adjusted threshold with *Q*-value = 0.05, which corresponds to an uncorrected *P*-value threshold of 0.047.

Figure 4 shows the correlation between the mean percentages of change in CV and CT and the degree of brain activation that fathers showed in response to pictures of their own baby compared with an unknown baby. As indicated, the mean percentages of change in CV and CT were negatively correlated with the fathers’ mean of the significant functional brain activation for the contrast “own baby> unknown baby” (*r*_CV_ = −0.55 and *r*_CT_ = −0.50; FDR-adjusted *P*-values = 0.047; Fig. 4). Specifically, the more reductions in CV and CT, the more the father’s brain activity in response to pictures of their baby compared with an unknown baby.

## Discussion

This is the first study presenting evidence for neuroanatomic changes—specifically, reductions in volume and thickness within default mode network brain structures—from preconception-to-postpartum in first-time fathers. These findings indicate that men undergo less pronounced—but still detectable and meaningful—neuroanatomic changes than mothers when transitioning into fatherhood for the first time. On the other hand, our results show that the observed changes are associated with the father’s brain response to cues of his infant, suggesting that such changes are related to the individual paternal sensitivity towards his baby.

Until now, there were only 2 structural MRI studies that followed men prospectively during their transition to fatherhood. In one of the studies, fathers were scanned at 2–4 weeks and later at 12–16 weeks postpartum, 44% of them being first-time fathers (Kim et al. 2014). The other study, published by our group, encompassed the pregnancy period by scanning first-time fathers before their partner’s pregnancies and at 10 weeks postpartum (Hoekzema et al. 2017). Although both studies used similar voxel-based morphometric approaches and captured overlapping times of the transition into fatherhood, their results were inconsistent. Although Kim et al. (2014) found significant gray matter volume changes between the 2 postpartum sessions, first-time fathers did not show significant brain changes between preconception and 10 weeks postpartum in Hoekzema et al. (2017). Hereby, we address these mixed results by reanalyzing Hoekzema *et al*. longitudinal MR images using a different approach consisting of surface-based morphometric methods and robust statistical methods based on permutations. We found that first-time fathers exhibit gray matter volume reductions and cortical thinning within the precuneus, a crucial node of the default mode network. These results are in line with Kim et al. (2014), who also found CV decreases within various default mode regions, including the precuneus. Notably, total CV, mean CT, and total CA in first-time fathers did not significantly differ from 0. However, group comparisons revealed no significant differences between fathers and mothers in any of the cortical measures. These findings suggest that fatherhood entails neuroanatomic changes that, as compared with those experienced by mothers, are less pronounced and affect fewer parts of the brain (Hoekzema et al. 2017; Carmona et al. 2019).

The precuneus is a core region of the default mode network. The default mode network is involved in the human ability to infer other people’s mental states, an ability commonly referred to as the theory of mind or mentalization, which facilitates empathy (Raichle et al. 2001). It has been suggested that parents engage in this ability to respond appropriately to their infant’s needs (Pavarini et al. 2013). Indeed, the default mode network belongs to the global parental caregiving system, which comprises several interconnected networks that support parental care (Feldman 2015). The precuneus, together with other default mode network regions, is commonly activated in fathers in response to their infants’ cues (Atzil et al. 2012; Mascaro et al. 2014; Li et al. 2018; Thijssen et al. 2018), which has also been observed in the current study. Our findings suggest that the neuroanatomic adaptations of men as they become fathers affect midline regions involved in their ability to understand the infants’ needs and respond accordingly.

Although cortical reductions sometimes reflect a process of neurodegeneration, they can also result from the refinement and specialization of neural circuits, a process mediated by synaptic pruning (Selemon 2013). Pruned synapses, together with a loss of surrounding glial, axonal, and vascular processes, are thought to underlie the cortical thinning observed during adolescence or other life stages of high neuroplasticity (Petanjek et al. 2011; Mills and Tamnes 2014). A previous study of our group found that pregnancy and adolescence exert similar morphometric effects on the human brain’s cortical mantle (Carmona et al. 2019). Indeed, both stages—pregnancy and adolescence—present associations between neuroanatomical reductions and brain activity. In adolescence, the more the cortical thinning, the stronger the brain activation in response to comprehension tasks (Lu et al. 2009; Nuñez et al. 2011). In pregnancy, the higher the volume reductions in the ventral striatum, a critical region of the parental reward system, the more it activates in response to offspring cues (Hoekzema et al. 2020). A very similar correlation between volume and thickness reductions and functional activity in the default mode network was observed in the current study. Therefore, considering previous literature, our findings suggest a neural specialization taking place during the transition into fatherhood rather than a neurodegenerative process. Future studies evaluating the brain at a cellular level are needed to elucidate the exact physiological mechanisms underlying our findings.

This study offers the first evidence of a relation between structural and functional brain changes in fatherhood. In humans, intersubject variability in paternal behavior is considerable: Paternal investment ranges from a father being completely missing to be the primary caregiver. The level of interaction with the infant, different sensitivity to hormones, social environment, or available resources for the infant upbringing are all factors that could influence the amount and quality of paternal behavior (Rajhans et al. 2019). In light of such behavioral variability, it is reasonable to think that the neuroanatomic adaptations accompanying fatherhood may depend on the amount of paternal investment. For instance, the brain volume changes found by Kim et al. (2014) were related to father-to-infant interactions: The higher the father’s physical contact with the infant during recorded interaction sessions, the higher the volume decreases in emotion regulation regions. We found that the more the volumetric reductions and cortical thinning in first-time fathers, the higher the brain’s response to pictures of their infants. This finding suggests that the neuroanatomic adaptations accompanying the transition into fatherhood are related to the individual response of the father to his infant.

The current study design does not allow for an analysis of the exact timing of these neuroanatomic changes. Do these changes initiate during the gestation of the baby, or is the postpartum environment period that triggers them? In females, nonhuman animal models suggest that the dramatic hormonal changes occurring during pregnancy and parturition are the main contributors to the neural remodeling underlying the onset of maternal behavior (Brunton and Russell 2015). Regarding males, there is substantial evidence that the continuous stimulation by pups after delivery progressively favors the paternal transition from attacking them to parenting them (Tachikawa et al. 2013). According to these data, the male’s brain adaptations to fatherhood would begin after parturition. However, there is scientific evidence that behavioral changes can be observed even before birth in certain species. For example, male rodents stop avoiding or attacking pups right before the delivery of their mated partner (Dulac et al. 2014). Whether brain changes occur before or after parturition in humans is still unknown. Given the human ability for imaging future events (Schacter and Addis 2007), it is likely that certain aspects of parental care can be simulated before the arrival of the newborn, thus triggering the remodeling of the paternal brain circuit. Indeed, men’s testosterone levels and their neural responses to infants’ stimuli are known to start changing before birth (Saxbe et al. 2017; Thijssen et al. 2018; Khoddam et al. 2020). Here, no significant correlations were found between morphological brain changes and the baby’s age, suggesting that these changes are not related to the duration of the postpartum father–infant experience. However, the baby’s age may not be the best indicator of the quality and duration of father-to-infant interactions: A father may have been absent during most of the postpartum period. Other measures, such as self-reported questionnaires or short father-to-infant interaction sessions, could help disentangle this question, though these tools may be affected by social desirability biases. Future studies with appropriate measures of paternal investment may help elucidate when these neuroanatomic adaptations are triggered.

The findings of this study should be considered in light of the limitations mentioned above. First, the study design does not allow for a more precise analysis of the timing of the brain adaptations for fatherhood. Second, available measurements are limited in capturing the amount and quality of paternal investment. Third, our analyses were limited to cortical regions: We did not provide information about potential changes in subcortical areas. Finally, given the variability in paternal behavior, our findings may still suffer from false negative results. That is, although we have tried to use statistical methods that optimize the balance between sensitivity and specificity, the precuneus may not be the only brain area that is associated with the transition to fatherhood. Despite these limitations, our results provide the first evidence that first-time fathers experience neuroanatomical adaptations that are associated with their brain responses towards their infants’ cues, suggesting an adaptive role of these brain changes for the challenges posed by fatherhood.

## Notes

The authors acknowledge the participants for their contribution in the current study. *Conflict of interest*: None declared.

## Funding

Ministerio de Ciencia, Innovación y Universidades project (RTI2018-093952-B-100); Instituto de Salud Carlos III projects (CP16/00096 and PI17/00064); cofunded by European Regional Development Fund, “A way of making Europe” and by “La Caixa” Foundation under the project code LCF/PR/HR19/52160001, and by the European Research Council under the project code 883069. The project ASPIDE has received funding from the European Union’s Horizon 2020 research and innovation programme under grant agreement number 801091. M.M.G. and S.S.C. were funded by Ministerio de Ciencia, Innovación y Universidades, Instituto de Salud Carlos III (PFIS contract FI18/00255 and Miguel Servet Type I research contract CP16/00096, respectively), and cofunded by European Social Fund “Investing in your future.” The Centro Nacional de Investigaciones Cardiovasculares is supported by the Ministerio de Ciencia, Innovación y Universidades and the Pro CNIC Foundation, and is a Severo Ochoa Center of Excellence (SEV-2015-0505). The funders had no role in study design, data collection and analysis, decision to publish, or preparation of the manuscript.

## Supplementary Material

Supplementary_Material_tgaa082

## References

[ref1] Abraham E, Hendler T, Shapira-Lichter I, Kanat-Maymon Y, Zagoory-Sharon O, Feldman R. 2014. Father’s brain is sensitive to childcare experiences. Proc Natl Acad Sci. 111:9792–9797. 24912146 10.1073/pnas.1402569111 PMC4103311

[ref2] Abraham E, Hendler T, Zagoory-Sharon O, Feldman R. 2016. Network integrity of the parental brain in infancy supports the development of children’s social competencies. Soc Cogn Affect Neurosci. 11:1707–1718. 27369068 10.1093/scan/nsw090 PMC5091682

[ref3] Atzil S, Hendler T, Zagoory-Sharon O, Winetraub Y, Feldman R. 2012. Synchrony and specificity in the maternal and the paternal brain: relations to oxytocin and vasopressin. J Am Acad Child Adolesc Psychiatry. 51:798–811. doi: 10.1016/j.jaac.2012.06.008. 22840551

[ref4] Benjamini Y, Hochberg Y. 1995. Controlling the false discovery rate: a practical and powerful approach to multiple testing. J R Stat Soc B Methodol. 57:289–300.

[ref5] Biffen SC, Warton CMR, Dodge NC, Molteno CD, Jacobson JL, Jacobson SW, Meintjes EM. 2020. Validity of automated FreeSurfer segmentation compared to manual tracing in detecting prenatal alcohol exposure-related subcortical and corpus callosal alterations in 9- to 11-year-old children. NeuroImage Clin. 28:102368. 32791491 10.1016/j.nicl.2020.102368 PMC7424233

[ref6] Brunton P, Russell J. Forthcoming 2015. Maternal brain adaptations in pregnancy. In: Plant TM, Zeleznik AJ editors. Knobil and Neill’s physiology of reproduction: two-volume set. 4th ed. Elsevier.

[ref7] Cabrera NJ, Tamis-LeMonda CS, Bradley RH, Hofferth S, Lamb ME. 2000. Fatherhood in the twenty-first century. Child Dev. 71:127–136. 10836566 10.1111/1467-8624.00126

[ref8] Carmona S, Martínez-García M, Paternina-Die M, Barba-Müller E, Wierenga LM, Alemán-Gómez Y, Pretus C, Marcos-Vidal L, Beumala L, Cortizo R, et al. 2019. Pregnancy and adolescence entail similar neuroanatomical adaptations: a comparative analysis of cerebral morphometric changes. Hum Brain Mapp. 40:2143–2152. 30663172 10.1002/hbm.24513 PMC6865685

[ref9] Doherty WJ, Kouneski EF, Erickson MF. 1998. Responsible fathering: an overview and conceptual framework. J Marriage Fam. 60:277.

[ref10] Dulac C, O’Connell LA, Wu Z. 2014. Neural control of maternal and paternal behaviors. Science. 345:765–770. 25124430 10.1126/science.1253291 PMC4230532

[ref11] Feldman R . 2015. The adaptive human parental brain: implications for children’s social development. Trends Neurosci. 38:387–399. 25956962 10.1016/j.tins.2015.04.004

[ref12] Feldman R, Braun K, Champagne FA. 2019. The neural mechanisms and consequences of paternal caregiving. Nat Rev Neurosci. 20:205–224. 30760881 10.1038/s41583-019-0124-6

[ref13] Gauthier AH, Smeeding TM, Furstenberg FF. 2004. Are parents investing less time in children? Trends in selected industrialized countries. Popul Dev Rev. 30:647–672.

[ref14] Geary DC . 2015. Evolution of paternal investment. In: Bus DM, editor. The handbook of evolutionary psychology. p. 483–505. 10.1002/9780470939376.ch16.

[ref15] Gettler LT, McDade TW, Feranil AB, Kuzawa CW. 2011. Longitudinal evidence that fatherhood decreases testosterone in human males. Proc Natl Acad Sci U S A. 108:16194–16199. 21911391 10.1073/pnas.1105403108 PMC3182719

[ref16] Gordon I, Zagoory-Sharon O, Leckman JF, Feldman R. 2010a. Prolactin, oxytocin, and the development of paternal behavior across the first six months of fatherhood. Horm Behav 58:513–518. 20399783 10.1016/j.yhbeh.2010.04.007 PMC3247300

[ref17] Gordon I, Zagoory-Sharon O, Leckman JF, Feldman R. 2010b. Oxytocin and the development of parenting in humans. Biol Psychiatry. 68:377–382. 20359699 10.1016/j.biopsych.2010.02.005 PMC3943240

[ref18] Greve DN, Fischl B. 2019. False positive rates in surface-based anatomical analysis. Neuroimage. 171:6–14. 10.1016/j.neuroimage.2017.12.072 PMC5857431 29288131

[ref19] Hoekzema E, Barba-Müller E, Pozzobon C, Picado M, Lucco F, García-García D, Soliva JC, Tobeña A, Desco M, Crone EA, et al. 2017. Pregnancy leads to long-lasting changes in human brain structure. Nat Neurosci. 20:287–296. 27991897 10.1038/nn.4458

[ref20] Hoekzema E, Tamnes CK, Berns P, Barba-Müller E, Pozzobon C, Picado M, Lucco F, Martínez-García M, Desco M, Ballesteros A, et al. 2020. Becoming a mother entails anatomical changes in the ventral striatum of the human brain that facilitate its responsiveness to offspring cues. Psychoneuroendocrinology. 112:104507. 31757430 10.1016/j.psyneuen.2019.104507

[ref21] Khoddam H, Goldenberg D, Stoycos SA, Horton KT, Marshall N, Cárdenas SI, Kaplan J, Saxbe D. 2020. How do expectant fathers respond to infant cry? Examining brain and behavioral responses and the moderating role of testosterone. Soc Cogn Affect Neurosci. 15:437–446. 10.1093/scan/nsaa051. 32307534 PMC7308657

[ref22] Kim P, Rigo P, Mayes LC, Feldman R, Leckman JF, Swain JE. 2014. Neural plasticity in fathers of human infants. Soc Neurosci. 9:522–535. doi: 10.1080/17470919.2014.933713. 24958358 PMC4144350

[ref23] Leibenluft E, Gobbini MI, Harrison T, Haxby JV. 2004. Mothers’ neural activation in response to pictures of their children and other children. Biol Psychiatry. 56:225–232. 15312809 10.1016/j.biopsych.2004.05.017

[ref24] Li T, Horta M, Mascaro JS, Bijanki K, Arnal LH, Adams M, Barr RG, Rilling JK. 2018. Explaining individual variation in paternal brain responses to infant cries. Physiol Behav. 193:43–54. 29730041 10.1016/j.physbeh.2017.12.033 PMC6015531

[ref25] Li W, Mai X, Liu C. 2014. The default mode network and social understanding of others: what do brain connectivity studies tell us. Front Hum Neurosci. 8:1–15. 24605094 10.3389/fnhum.2014.00074 PMC3932552

[ref26] Lu LH, Dapretto M, O’Hare ED, Kan E, McCourt ST, Thompson PM, Toga AW, Bookheimer SY, Sowell ER. 2009. Relationships between brain activation and brain structure in normally developing children. Cereb Cortex. 19:2595–2604. 19240138 10.1093/cercor/bhp011 PMC2758677

[ref27] Malmberg LE, Lewis S, West A, Murray E, Sylva K, Stein A. 2016. The influence of mothers’ and fathers’ sensitivity in the first year of life on children’s cognitive outcomes at 18 and 36 months. Child Care Health Dev. 42:1–7. 26538379 10.1111/cch.12294

[ref28] Mascaro JS, Hackett PD, Gouzoules H, Lori A, Rilling JK. 2014. Behavioral and genetic correlates of the neural response to infant crying among human fathers. Soc Cogn Affect Neurosci. 9:1704–1712. 24336349 10.1093/scan/nst166 PMC4221211

[ref29] Mills KL, Tamnes CK. 2014. Methods and considerations for longitudinal structural brain imaging analysis across development. Dev Cogn Neurosci. 9:172–190. 24879112 10.1016/j.dcn.2014.04.004 PMC6989768

[ref30] Noble S, Scheinost D, Constable RT. 2020. Cluster failure or power failure? Evaluating sensitivity in cluster-level inference. Neuroimage 209:116468. 31852625 10.1016/j.neuroimage.2019.116468 PMC8061745

[ref31] Nuñez SC, Dapretto M, Katzir T, Starr A, Bramen J, Kan E, Bookheimer S, Sowell ER. 2011. fMRI of syntactic processing in typically developing children: structural correlates in the inferior frontal gyrus. Dev Cogn Neurosci. 1:313–323. 21743820 10.1016/j.dcn.2011.02.004 PMC3129989

[ref32] O’Brien M, Brandth B, Kvande E. 2007. Fathers, work and family life. Community Work Fam. 10:375–386.

[ref33] Ozgun O, Erden S, Ciftci MA. 2011. Examining different perspectives on fatherhood: a socio-cultural approach. Procedia Soc Behav Sci. 15:364–368.

[ref34] Pavarini G, de Hollanda-Souza D, Hawk CK. 2013. Parental practices and theory of mind development. J Child Fam Stud. 22:844–853.

[ref35] Petanjek Z, Judas M, Simic G, Rasin MR, Uylings HBM, Rakic P, Kostovic I. 2011. Extraordinary neoteny of synaptic spines in the human prefrontal cortex. Proc Natl Acad Sci. 108:13281–13286. 21788513 10.1073/pnas.1105108108 PMC3156171

[ref36] Raichle ME, MacLeod AM, Snyder AZ, Powers WJ, Gusnard DA, Shulman GL. 2001. A default mode of brain function. Proc Natl Acad Sci U S A. 98:676–682. 11209064 10.1073/pnas.98.2.676 PMC14647

[ref37] Rajhans P, Goin-Kochel RP, Strathearn L, Kim S. 2019. It takes two! Exploring sex differences in parenting neurobiology and behaviour. J Neuroendocrinol. 31:e12721. 10.1111/jne.12721. PMC6773483 31034670

[ref38] Reuter M, Fischl B. 2011. Avoiding asymmetry-induced bias in longitudinal image processing. Neuroimage 57:19–21. 21376812 10.1016/j.neuroimage.2011.02.076 PMC3260043

[ref39] Reuter M, Rosas HD, Fischl B. 2010. Highly accurate inverse consistent registration: a robust approach. Neuroimage. 53:1181–1196. 20637289 10.1016/j.neuroimage.2010.07.020 PMC2946852

[ref40] Reuter M, Schmansky NJ, Rosas HD, Fischl B. 2012. Within-subject template estimation for unbiased longitudinal image analysis. Neuroimage. 61:1402–1418. 22430496 10.1016/j.neuroimage.2012.02.084 PMC3389460

[ref41] Rohner RP . 1998. Father love and child development: history and current evidence. Curr Dir Psychol Sci. 7:157–161.

[ref42] Sarkadi A, Kristiansson R, Oberklaid F, Bremberg S. 2008. Fathers’ involvement and children’s developmental outcomes: a systematic review of longitudinal studies. Acta Paediatr. 97:153–158. 18052995 10.1111/j.1651-2227.2007.00572.x

[ref43] Saxbe D, Rossin-Slater M, Goldenberg D. 2018. The transition to parenthood as a critical window for adult health. Am Psychol. 73:1190–1200. 30525801 10.1037/amp0000376

[ref44] Saxbe DE, Edelstein RS, Lyden HM, Wardecker BM, Chopik WJ, Moors AC. 2017. Fathers’ decline in testosterone and synchrony with partner testosterone during pregnancy predicts greater postpartum relationship investment. Horm Behav. 90:39–47. 27469070 10.1016/j.yhbeh.2016.07.005

[ref45] Sayer LC, Bianchi SM, Robinson JP. 2004. Are parents investing less in children? Trends in mothers’ and fathers’ time with children. Am J Sociol. 110:1–43.

[ref46] Schacter DL, Addis DR. 2007. The cognitive neuroscience of constructive memory: remembering the past and imagining the future. Philos Trans R Soc B Biol Sci. 362:773–786. 10.1098/rstb.2007.2087 PMC2429996 17395575

[ref47] Selemon LD . 2013. A role for synaptic plasticity in the adolescent development of executive function. Transl Psychiatry. 3:e238. 10.1038/tp.2013.7. PMC3625918 23462989

[ref48] Smith SM, Nichols TE. 2009. Threshold-free cluster enhancement: addressing problems of smoothing, threshold dependence and localisation in cluster inference. Neuroimage 44:83–98. 18501637 10.1016/j.neuroimage.2008.03.061

[ref49] Swain JE . 2008. Baby stimuli and the parent brain: functional neuroimaging of the neural substrates of parent-infant attachment. Psychiatry (Edgmont). 5:28–36. PMC2695737 19727273

[ref50] Tachikawa KS, Yoshihara Y, Kuroda KO. 2013. Behavioral transition from attack to parenting in male mice : a crucial role of the vomeronasal system. 33:5120–5126. 10.1523/JNEUROSCI.2364-12.2013 PMC6705007 23516278

[ref51] Thijssen S, Van ‘t Veer AE, Witteman J, Meijer WM, van IJzendoorn MH, Bakermans-Kranenburg MJ. 2018. Effects of vasopressin on neural processing of infant crying in expectant fathers. Horm Behav. 103:19–27. 29792885 10.1016/j.yhbeh.2018.05.014

[ref52] Winkler AM, Ridgway GR, Webster MA, Smith SM, Nichols TE. 2014. Permutation inference for the general linear model. Neuroimage. 92:381–397. 24530839 10.1016/j.neuroimage.2014.01.060 PMC4010955

[ref53] Woodroffe R, Vincent A. 1994. Mother’s little helpers: patterns of male care in mammals. Trends Ecol Evol. 9:294–297. 21236858 10.1016/0169-5347(94)90033-7

[ref54] Yeo BTT, Krienen FM, Sepulcre J, Sabuncu MR, Lashkari D, Hollinshead M, Roffman JL, Smoller JW, Zöllei L, Polimeni JR, et al. 2011. The organization of the human cerebral cortex estimated by intrinsic functional connectivity. J Neurophysiol. 106:1125–1165. 21653723 10.1152/jn.00338.2011 PMC3174820

