# Supplementary material for: The Paternal Transition Entails Neuroanatomic Adaptations that are Associated with the Father’s Brain Response to his Infant Cues
Source: Cereb Cortex Commun. 2020 Nov 4;1(1):tgaa082. doi: 10.1093/texcom/tgaa082 (PMC8152902; doi:10.1093/texcom/tgaa082)
Supplement: Supplementary_Material_tgaa082 [file supplementary_material_tgaa082.docx]

**SUPPLEMENTARY MATERIAL**

**Supplementary Table 1:** Basic descriptive and inferential statistics of within and between-group comparisons, and bootstrap distributions of the percentages of change in total cortical volume, mean cortical thickness, and total cortical area.

|  |  | **Within-group comparison** | | | **Between-group comparison** | | |
| --- | --- | --- | --- | --- | --- | --- | --- |
| **Measure**  **(% of change)** | **Statistic** | **First-time Fathers** | **Childless Men** | **First-time Mothers** | **First-time Fathers vs. First-time Mothers** | **First-time Fathers vs. Childless Men** | **First-Time Mothers vs Childless Men** |
| **Total**  **Cortical Volume** | **Mean (s.d)** | -0.897 (2.661) | 0.143 (2.240) | -2.884 (2.941) | × | × | × |
|  | **T-statistic** | -1.508 | 0.263 | -4.903 | 2.348 | -1.272 | -3.590 |
|  | **Unc. p-value** | 0.148 | 0.796 | 0.0001 | 0.024 | 0.212 | 0.0009 |
|  | **FDR-adj. p-value** | 0.333 | 0.903 | 0.0005 | 0.053 | 0.272 | 0.0081 |
|  | **Effect size** | -0.337 | 0.064 | -0.981 | 0.716 | -0.430 | -1.135 |
|  | **Bootstrap mean (s.d)** | -0.8963 (0.5840) | 0.1415 (0.5292) | -2.8861 (0.5798) | × | × | × |
|  | **95% bootstrap CI** | -2.0783; 0.2020 | -0.7337; 1.3361 | -4.0962; -1.8249 | × | × | × |
| **Mean**  **Cortical Thickness** | **Mean (s.d)** | -0.748 (2.455) | 0.132 (2.144) | -2.002 (2.465) | × | × | × |
|  | **T-statistic** | -1.362 | 0.254 | -4.061 | 1.699 | -1.151 | -2.899 |
|  | **Unc. p-value** | 0.189 | 0.803 | 0.0005 | 0.096 | 0.258 | 0.006 |
|  | **FDR-adj. p-value** | 0.340 | 0.903 | 0.002 | 0.145 | 0.290 | 0.027 |
|  | **Effect size** | -0.305 | 0.062 | -0.812 | 0.518 | -0.389 | -0.917 |
|  | **Bootstrap mean (s.d)** | -0.7468 (0.5355) | 0.1324 (0.4992) | -2.0131 (0.4796) | × | × | × |
|  | **95% bootstrap CI** | -1.9006; 0.1748 | -0.8257; 1.1308 | -2.9809; -1.0949 | × | × | × |
| **Total**  **Cortical Area** | **Mean (s.d)** | -0.177 (0.960) | 0.003 (0.779) | -0.950 (1.297) | × | × | × |
|  | **T-statistic** | -0.826 | 0.018 | -3.663 | 2.220 | -0.622 | -2.711 |
|  | **Unc. p-value** | 0.419 | 0.986 | 0.001 | 0.032 | 0.538 | 0.010 |
|  | **FDR-adj. p-value** | 0.628 | 0.986 | 0.004 | 0.0572 | 0.538 | 0.029 |
|  | **Effect size** | -0.1848 | 0.004 | -0.733 | 0.677 | -0.210 | -0.857 |
|  | **Bootstrap mean (s.d)** | -0.1784 (0.2082) | 0.0038 (0.1831) | -0.9523 (0.2532) | × | × | × |
|  | **95% bootstrap CI** | -0.5413; 0.2926 | -0.3730; 0.3398 | -1.7306; -0.6057 | × | × | × |
| The FDR-adjusted thresholds at a q-value of 0.05 for the within-group comparisons and the between-group comparison correspond to a p-value of 0.001 and 0.010, respectively.  Abbreviations are as follows: s.d= standard deviation, FDR= False Discovery Rate, unc.= uncorrected, adj.= adjusted, and CI= Confidence Interval. | | | | | | | |


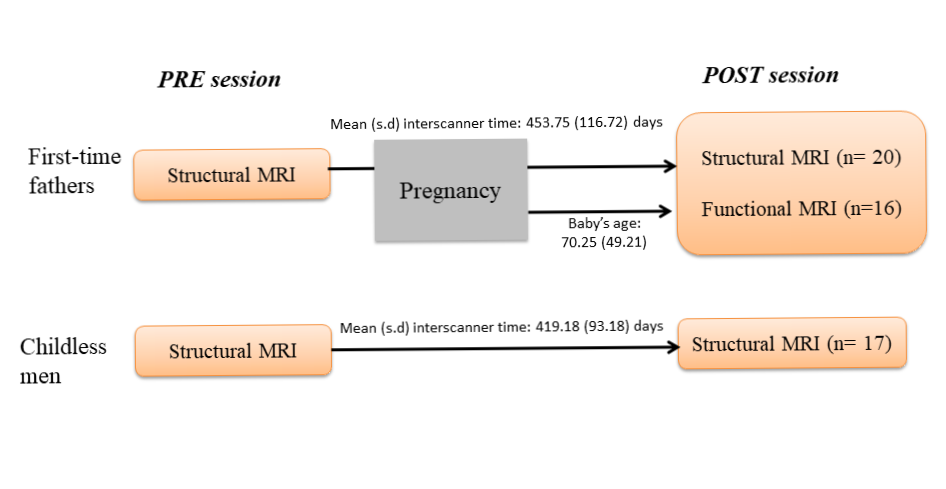


**Supplementary Figure 1: Schematic representation of the prospective multimodal study design.** First-time fathers were scanned before the pregnancy of their partners (PRE session) and again during the postpartum (POST session). Childless men were scanned at a comparable time interval. Abbreviation is s.d= standard deviation.

**
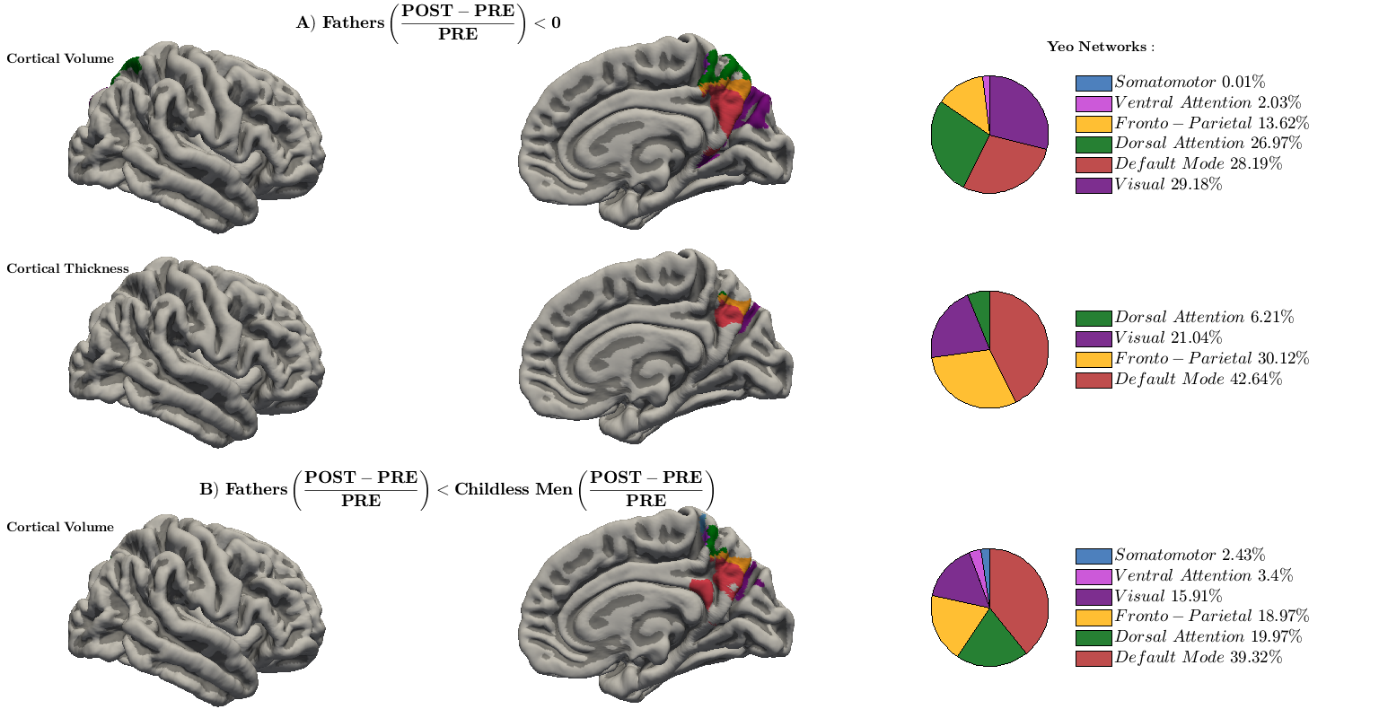
**

**Supplementary Fig 2: Surface-based cortical changes belonging to the seven functional networks of Yeo et al. (2011) in the right hemisphere. (A)** Reductions in first-time fathers relative to the baseline (preconception session), and **(B)** Greater volumetric reductions in first-time fathers compared to childless men. Represented results survive the restrictive threshold of TFCE, p-value< 0.05 FWE-corrected. Each network is depicted in a different color. Brain surfaces correspond to the pial fsaverage template of FreeSurfer. Abbreviations are as follows: PRE= preconception and POST= postpartum.

**Supplementary Methods and Results: Analyses accounting for the effect of radiofrequency head coil**

Due to an unexpected problem in the postpartum session, the 8-channel radiofrequency head coil had to be replaced by one of 16 channels in nine out of thirty-seven structural MR images (seven first-time fathers and two childless men, and Fisher’s exact test revealed that proportions are not significantly different; p-value= 0.137) and in five out of sixteen functional MR images. This replacement also affected the mothers’ group; ten out of twenty-five images were acquired with the latter coil. Thus, to err on the side of caution, we repeated the analyses using the head coil as an additional covariate.

*Global cortical structural changes*

The global analyses were repeated fitting a General Linear Model per cortical measure --total cortical volume, mean cortical thickness, and total cortical area--, including as covariates not only the group membership and the age z-scores but also the radiofrequency head coil for each subject. Then, we calculated the within-group and between-group comparisons, and the bootstrap distributions using the residuals of the percentage of change.

As indicated in Supplementary Fig 3, the results taking into account the potential variability induced by the head coil are similar to those obtained in the main analyses (Fig 1).


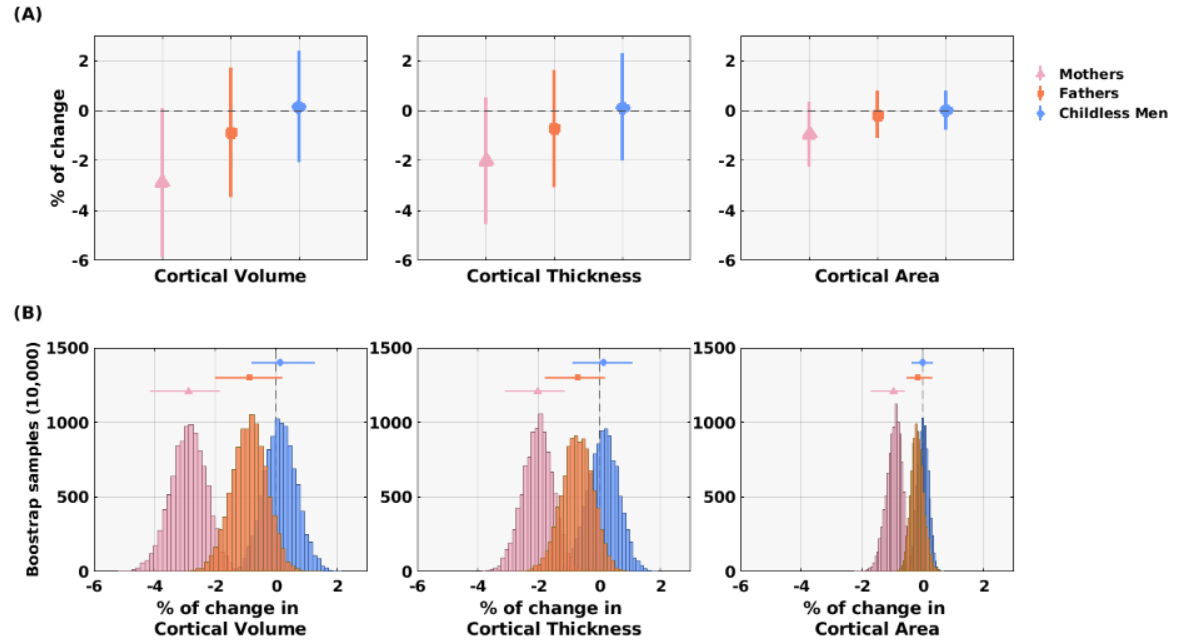


**Supplementary Fig 3: Global cortical percentages (%) of change in first-time mothers (N=25), first-time fathers (N=20), and childless men (N=17), accounting for possible head coil effects. (A)** The Y-axis represents the mean of the percentages of change. The three cortical measures --Total Cortical Volume, Mean Cortical Thickness, and Total Cortical Area-- are indicated in the X-axis. Vertical dispersion bars and the symbols represent the standard deviations and the mean values, respectively. **(B)** Bootstrap histograms of the mean percentage of change of each cortical measure. The Y-axis indicates the number of bootstrap samples (10,000 random samples), and the X-axis the mean total percentages of change of each bootstrap sample. Horizontal dispersion bars and the symbols represent the 95% confidence intervals and the mean of each bootstrap distribution, respectively. First-time mothers were included for visualization and comparative purposes.

*Surface-based Morphometry*

As performed in the main manuscript, we estimated the vertex-wise contrasts in PALM, this time including as an additional covariate the radiofrequency head coil. Also, we computed the percentage of overlap between the resulting structural surface-based changes and the seven Yeo functional networks (Thomas Yeo et al. 2011).

As indicated in Supplementary Fig 4, the surface-based changes in first-time fathers were located at the same anatomical regions as those reported in the main manuscript (Fig 2). Also, most of the within-group findings still fell into the default mode network. The exact percentages of overlap are depicted in Supplementary Fig 5.

Regarding the between-group comparisons, no significant volumetric differences were found between fathers and childless males. This could be attributed to the decrease in the degrees of freedom and the increase in the variance component --coming from the childless men-- which resulted in a lack of detection power. In fact, when we relaxed the statistical threshold to TFCE, FWE-corrected p-value<0.1, the same anatomical regions as those reported in the main manuscript appeared statistically significant (Supplementary Fig 4B).


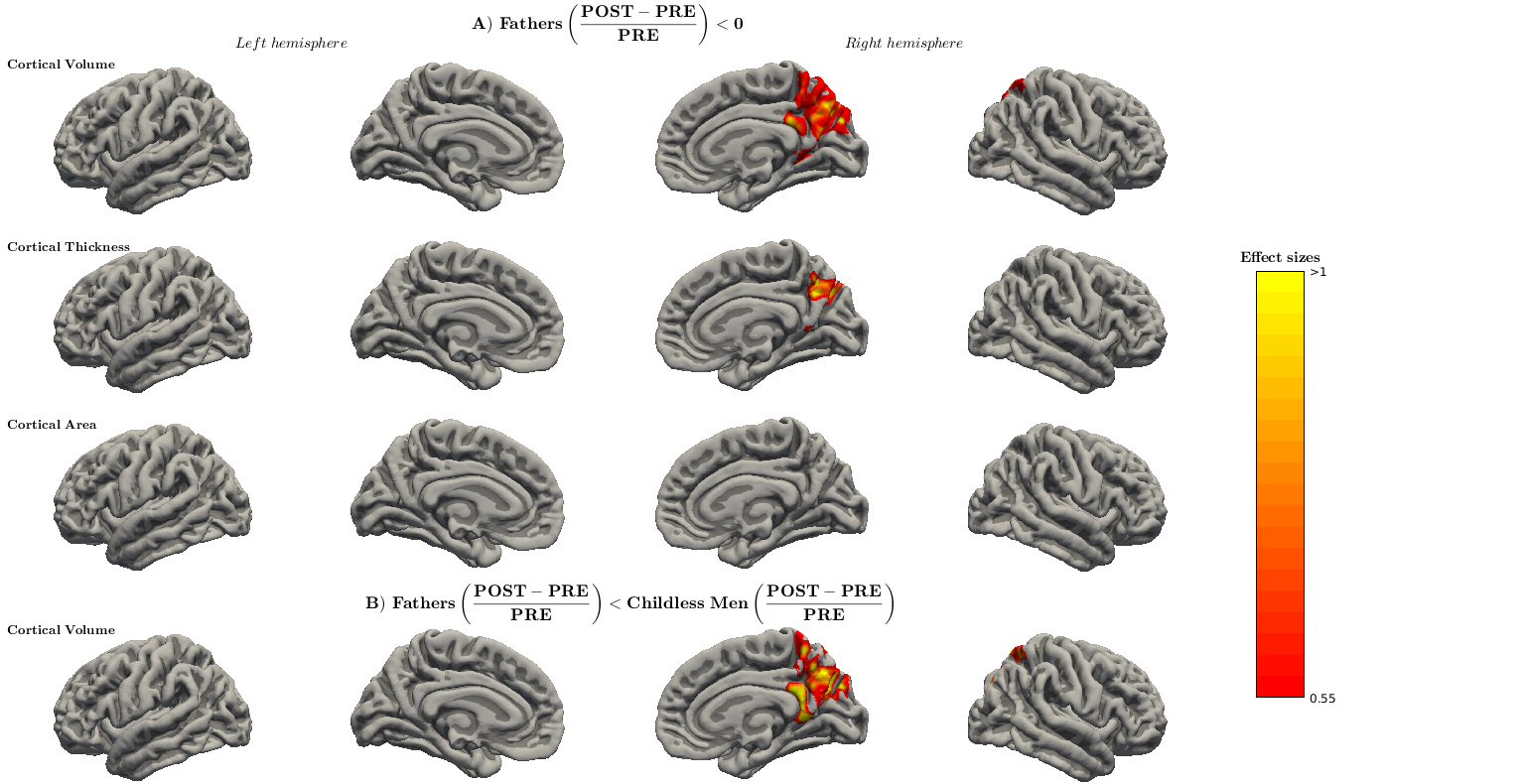


**Supplementary Fig 4:** S**urface-based cortical changes in first-time fathers (N= 20) and between fathers and childless men (N= 17) groups, accounting for possible head coil effects. (A)** Reductions in first-time fathers relative to baseline (preconception session) at the restrictive threshold of TFCE, p-value< 0.05 FWE-corrected. Cohen’s d effect sizes range between 0.56 and 1.10 for cortical volume and between 0.70 and 1.11 for cortical thickness. **(B)** Greater volumetric reductions in first-time fathers compared to childless men. Effect sizes range from 0.80 to 1.48 at the lenient threshold of TFCE, p-value< 0.1 FWE-corrected. Brain surfaces correspond to the pial fsaverage template of FreeSurfer. Abbreviations are as follows: PRE= preconception and POST= postpartum.

**
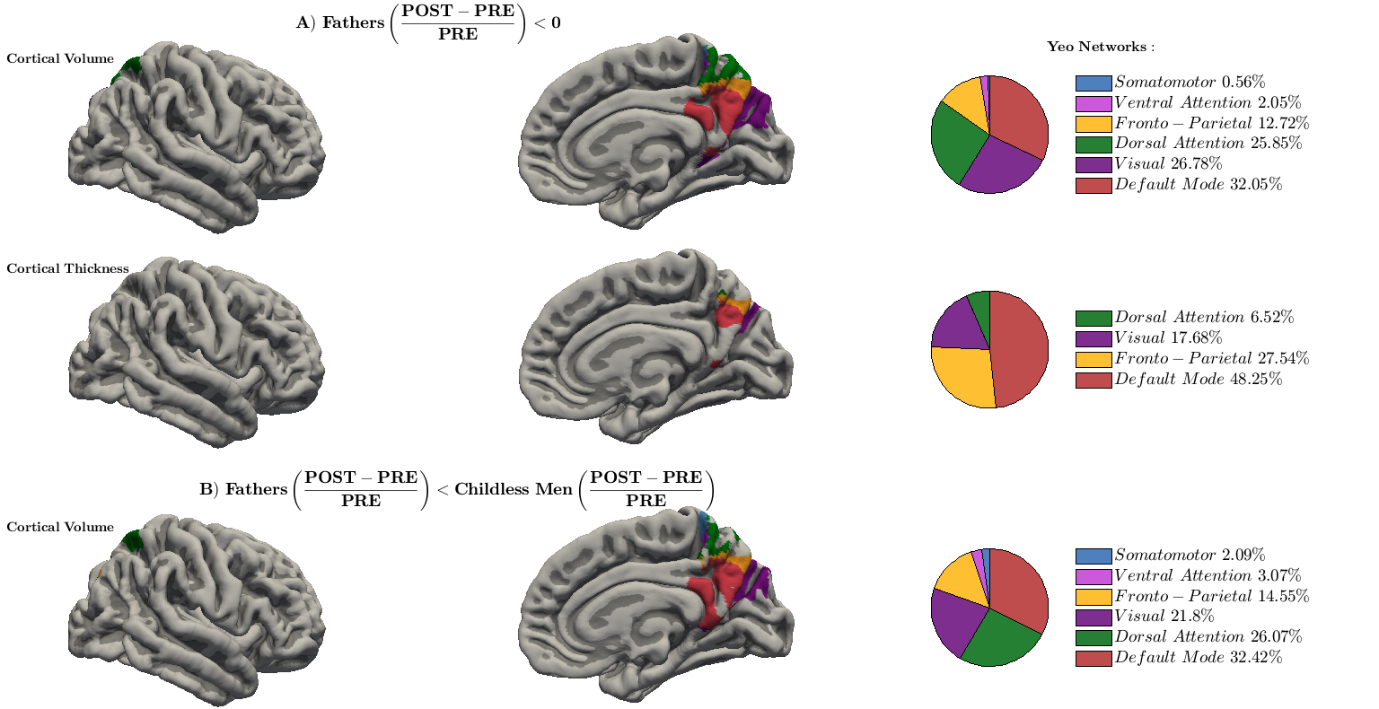
**

**Supplementary Fig 5: Surface-based cortical changes belonging to the seven functional networks of Yeo et al. (2011) in the right hemisphere, accounting for possible head coil effects. (A)** Reductions in first-time fathers relative to the baseline (preconception session) at the restrictive threshold of TFCE, p-value< 0.05 FWE-corrected, and **(B)** Greater volumetric reductions in first-time fathers compared to childless males at the lenient threshold of TFCE, p-value< 0.1 FWE-corrected. Each network is depicted in a different color and the brain surfaces correspond to the pial fsaverage template of FreeSurfer. Abbreviations are as follows: PRE= preconception and POST= postpartum.

*Correlations between cortical changes and indicators of paternal experience*

We re-calculated the correlations between the mean morphometric percentages of change in CV and CT and variables of paternal experience: the accumulated time that fathers had spent with their babies (as estimated by baby’s age), 2) the degree of brain activation that fathers showed upon their babies’ pictures as compared to an unknown baby’ pictures (as estimated by mean functional brain activations from Supplementary Fig 6), or 3) both. To obtain the morphological brain changes, we used the results of the surface-based morphometric analysis while controlling for the age and head coil effects (Supplementary Fig 4).

1. *Baby’s age*

No significant correlations were found between the morphological brain changes and the baby’s age (r_CV_ and r_CT_= 0.07 and 0.02; uncorrected p-values> 0.777).

1. *Degree of brain activation to own baby vs. unknown baby*

To obtain the degree of brain activation, we repeated the second-level analysis of the functional images. The model re-examined whether the neural activity in first-time fathers differed when watching pictures of their baby as compared to an unknown baby. It included as covariates the age z-scores and the radiofrequency head coil. Additionally, we calculated the percentage of overlap of the resulting functional brain activations and the seven functional networks of Yeo et al. (Thomas Yeo et al. 2011).

As indicated in Supplementary Fig 5, the neural responses of first-time fathers are located at the same anatomical regions as those reported in the main manuscript (Fig 3). Also, most of the significant functional brain activations still fell into the default mode and dorsal attention networks (62.03% and 19.5% of the total activations, respectively; Supplementary Fig 6B). As indicated in the results of the main manuscript, the reverse contrast (“unknown baby> own baby”) did not render any significant activations.


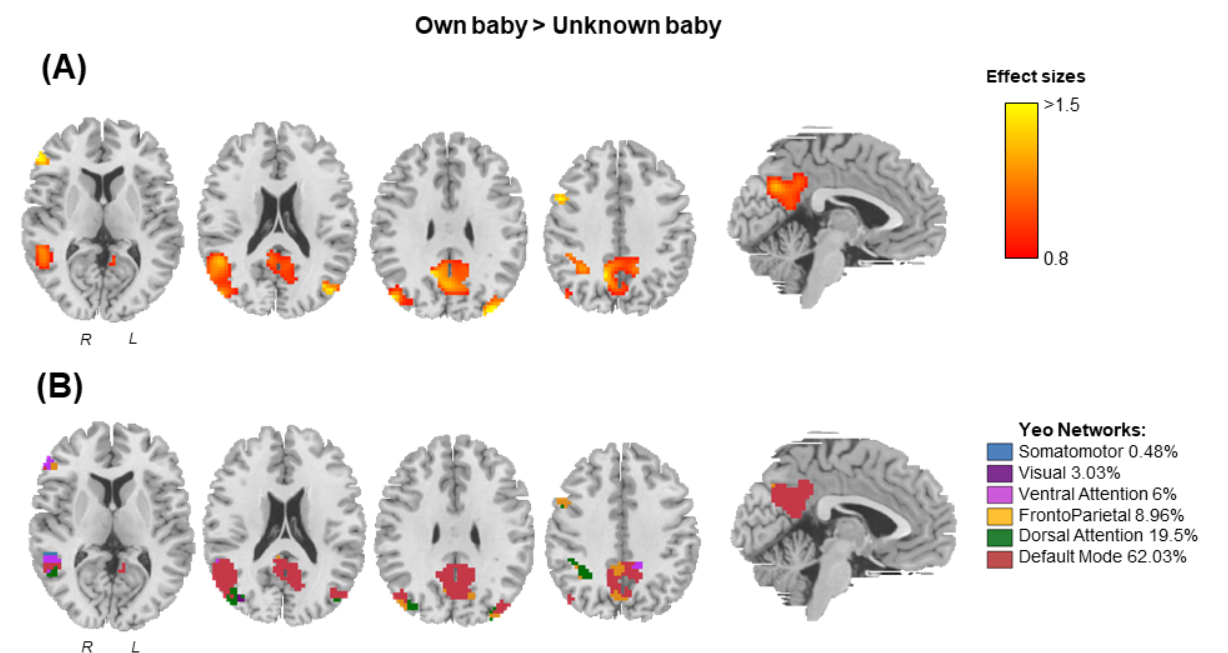


**Supplementary Fig 6: Functional brain activations in first-time fathers (N=16) for the contrast “own baby> unknown baby”, accounting for possible head coil effects. (A)** Effect sizes (Cohen’s d) of the functional activations, ranging from 0.86 to 1.82. **(B)** Percentage of overlap between the functional brain activations and each of the seven functional networks of Yeo et al. (2011). Each network is depicted in a different color. Results surviving the restrictive threshold of TFCE, p-value< 0.05 FWE-corrected. Slices correspond to the axial views (MNI coordinates: 9, 21, 30, and 39) and the sagittal view (MNI coordinate: -3). Abbreviations are as follows: MNI= Montreal Neurological Institute, L= Left hemisphere, and R= Right hemisphere.

Both the direction of the correlations and the associated statistical significance remained identical after the inclusion of the additional head coil covariate (Supplementary Fig 7). That is, negative correlations were found between the mean percentages of change in CV and CT and the mean of the significant fathers’ functional brain activation for the contrast “own baby> unknown baby” (r_CV_= -0.55 and r_CT_= -0.50, and FDR-adjusted p-values= 0.049).

**
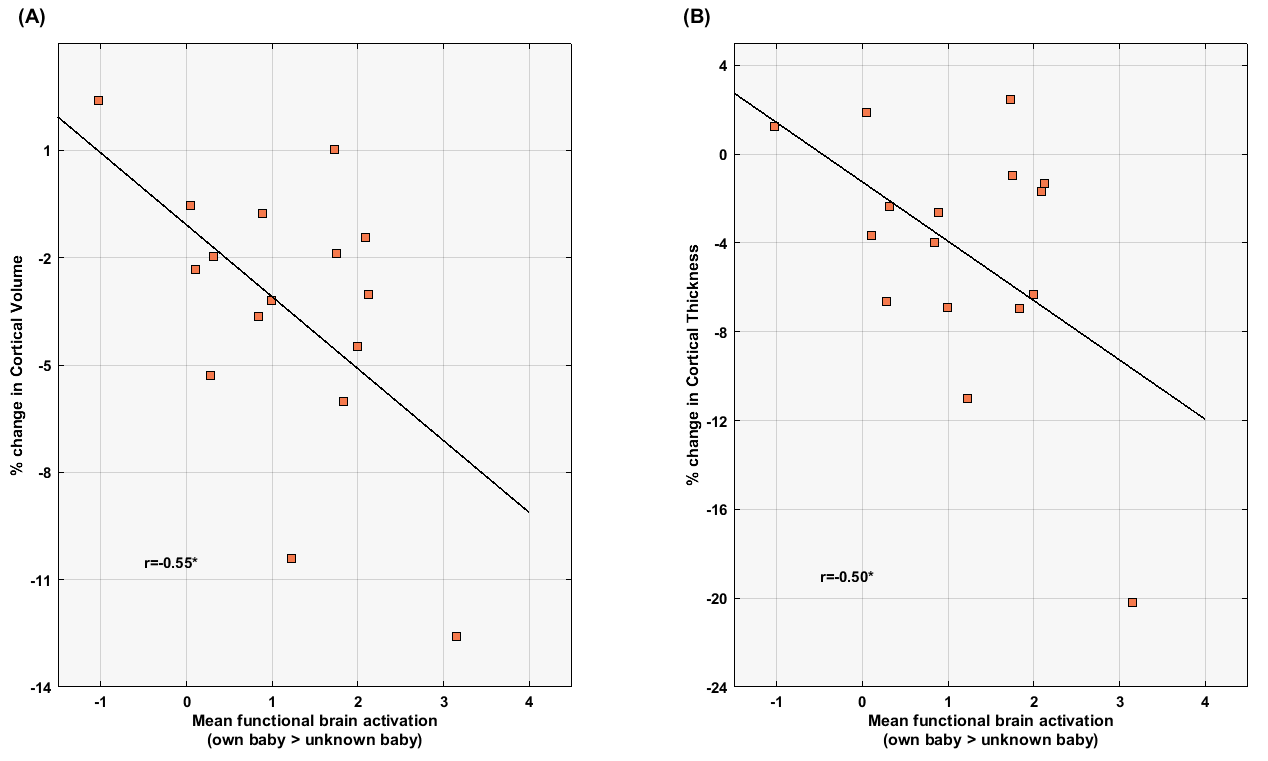
**

**Supplementary Fig 7: Correlations between cortical changes and functional brain activations in first-time fathers (N= 16).** The X-axis represents the mean of the significant functional brain activations towards pictures of their own baby compared to an unknown baby. The Y-axis represents the mean percentages of change in Cortical Volume **(A)** and Cortical Thickness **(B)** extracted from the surface-based morphometric analysis. Asterisks indicate the Pearson correlations coefficients (r) that survived the False Discovery Rate-adjusted threshold with Q-value= 0.05, which corresponds to an uncorrected p-value threshold of 0.049.
